# A Review of Early Childhood Caries: Risk Factors, Management, and Policy Recommendations

**DOI:** 10.7759/cureus.83767

**Published:** 2025-05-09

**Authors:** Niyomi S Patel, Miral Mehta, Yihan Fu, Vishnu Desai, Hetvi S Lala, Het Parikh, Mansi M Patel, Urja B Thakor

**Affiliations:** 1 Department of Pedodontics and Preventive Dentistry, Karnavati School of Dentistry, Karnavati University, Gandhinagar, IND; 2 Department of General Dentistry, SmileBuilderz, Lancaster, USA; 3 Department of Periodontology and Implantology, Karnavati School of Dentistry, Karnavati University, Gandhinagar, IND

**Keywords:** early childhood caries, fluoride, management, pediatric oral health, pediatric preventive dentistry

## Abstract

Initially, the American Academy of Pedodontics and the American Academy of Pediatrics (AAP) jointly addressed severe tooth decay related to bottle use by issuing a statement on Nursing Bottle Caries. However, over the next two decades, the American Academy of Pediatric Dentistry (AAPD) expanded its understanding, recognising that early childhood caries (ECC) resulted from various inadequate feeding practices, leading to the adoption of the broader term "ECC" to better reflect its complex causes. This review discusses ECC: its prevalence, risk factors, effects, prevention, early detection, and treatment. It emphasises diet, oral hygiene, socio-economic status, and the importance of parental education and access to dental services in ECC. The aim is to synthesise research to improve ECC understanding and management. This paper reviews the causes and effects of ECC on oral health. Data were obtained from databases like PubMed and Google Scholar, using relevant keywords. The review used English-language articles, including research and policies. This review reveals that ECC is a complex disease causing severe tooth decay in young children. ECC development is influenced by diet, oral hygiene, quality of life, fluoride intake, oral microbiota, environmental factors, and feeding practices. It can lead to short- and long-term issues, like pain, infection, eating and speech difficulties, poor development, and enamel defects. Effective prevention requires multifaceted strategies involving home care, dental interventions, community support, and policies. A major, yet preventable, disease, ECC significantly impacts global oral health, particularly in deprived children. Starting with the first tooth, it harms development and well-being. Prevention relies on public education, interprofessional collaboration, and implementing AAPD policy recommendations.

## Introduction and background

Dental caries is the most common preventable, noncommunicable disease affecting the calcified tissue of teeth [1]. It is characterised by the destruction of the organic structure and the demineralisation of the inorganic structure [2]. Dental caries develops when the biofilm microbiota, which typically exists in a balanced state in the oral cavity, transforms into an acid-producing, acid-tolerant, and cavity-causing population as a result of regular sugar intake [3].

In 1978, the American Academy of Pedodontics, alongside the American Academy of Pediatrics (AAP), issued a joint statement on Nursing Bottle Caries to address a severe form of dental caries linked to bottle use. In the following two decades, the American Academy of Pediatric Dentistry (AAPD) acknowledged that early childhood caries (ECC) was not solely linked to inadequate feeding practices, leading to the adoption of the term ECC to more accurately represent its complex aetiology [4,5]. Contributing factors include vulnerable teeth due to enamel hypoplasia, high levels of cariogenic bacteria in the mouth (notably *Mutans streptococci*), and the conversion of sugars by bacteria adhering to teeth, resulting in acid production that gradually demineralises tooth structure [4].

According to Davies (1998), ECC is defined as a complex disease involving maxillary primary incisors within a month after eruption, and spreading rapidly to other primary teeth [5]. This was later revised by the American Academy in 2002, which defined ECC as the presence of one or more decayed (non-cavitated or cavitated lesions), missing (due to caries), or filled tooth surfaces in any primary tooth in a child 71 months of age or younger. In children younger than three years, any sign of smooth-surface caries is indicative of severe early childhood caries (S-ECC). In children aged 3-5 years, one or more cavitated, missing, or filled smooth surfaces in the primary maxillary anterior teeth are considered severe ECC [6].

The occurrence of ECC is not caused by a single factor; rather, it results from multiple factors, such as high sugar consumption, low-fibre diet, developmental defects, high levels of *Streptococcus mutans* in the oral cavity, and socio-economic disadvantage. The prevalence of ECC can be reduced by implementing various strategies that focus on children's dietary habits, good oral hygiene, and the education of parents and caregivers regarding preventive and treatment options. ECC impacts both the child’s health and their quality of life [7].

Problem statement of this paper

ECC is an extensive dental problem among young children, causing severe tooth decay and irreversible oral health challenges. Poor eating habits, high sugar consumption, poor oral hygiene, and low access to dental services are the main causes of ECC, especially in low-income populations. Despite its preventability, ECC remains prevalent because there is low awareness among caregivers and limited early dental interventions. ECC not only affects a child's mouth, but may also lead to speech, dietary, and psychosocial complications, requiring an urgent focus to enhance prevention, early detection, and treatment plans for this disorder.

Objectives

This review article aims to discuss the prevalence, risk factors, and effects of ECC. It also intends to explore current research on preventive strategies, early detection, and effective treatment modalities for ECC. The article will emphasise contributing factors such as diet, oral hygiene, and socio-economic status in the aetiology of ECC, as well as the importance of parental education and access to dental services. By synthesising recent research findings, the article aims to enhance our understanding and inform future action in ECC prevention and management.

## Review

Methodology

The paper reviews the factors responsible for causing ECC and its effects on oral health. PubMed, Google Scholar, ResearchGate, and ScienceDirect databases were accessed to obtain relevant data. Search keywords such as "ECC", "dental decay", "oral health", "pediatric dentistry", "diagnosis", "risk factors", "prevention", "fluoride", "diet", and "oral hygiene" were used for inclusion.

An independent team of three researchers selected all the papers to avoid bias. This review included original research articles, systematic reviews, policies, and meta-analyses from English-language literature published between 2000 and 2024. The reference lists of these articles were also examined to identify additional relevant studies. The review was limited to all types of human studies and clinical trials, and excluded articles that were not related to ECC or were published in languages other than English.

Epidemiology

Dental caries is still a major problem in various developing and developed countries [8]. The prevalence of ECC is associated with a wide range of factors, such as low socioeconomic status, race, culture, ethnicity, inaccurate tooth brushing habits, lifestyle, dietary habits, and frequency of sugar intake. It also varies from country to country [9,10]. According to systematic review reports involving 67 countries, the prevalence rate in underdeveloped countries is as high as 70%, while the prevalence rate in most developed countries ranges between 1% and 12% [11]. Globally, 514 million children suffer from ECC. The worldwide estimated random-effects pooled prevalence of ECC stands at 49%. The random-effects pooled caries prevalence (ECC) was 34% (Central/South America), 36% (Europe), 42% (Africa), 52% (Asia-Oceania), 57% (North America), and 72% (Middle East) [12]. Worldwide, the highest ECC prevalence was reported in the Philippines (98.0%) [13], while the lowest was in Japan (20.6%) [14] and Greece (19.3%) [13]. In Asia, particularly in the Far East, the occurrence of the disease among three-year-olds varies from 36% to 85% [15]. The overall prevalence rate in India is 54.16%, and 57% among the 3- to 18-year-old population, according to a systematic review and meta-analysis published in 2021 [16]. The decrease in prevalence rate is attributed to increased awareness of oral hygiene as age advances. Depending on the location, there is a 58.9% occurrence rate of dental caries in the urban population, compared to 51.4% in the rural population. The high occurrence rate is due to the availability of junk food items rich in refined sugar and lower consumption of fibre-rich foods in their diet [17]. The prevalence rate in the low socioeconomic status group was 61.8% [17]. In accordance with findings presented at the International Association of Pediatric Dentistry (IAPD) Conference in 2018, the prevalence rate of ECC among children aged 1-5 years was 17%, 36%, 43%, 55%, and 63%, respectively, indicating that the rate escalates with increasing age [18]. 

Etiology

Dental caries is an infectious and multifaceted disease influenced by several etiological factors that contribute to its initiation and progression [19]. The condition develops through the simultaneous interaction of at least three key elements, known as Keyes’ triad [20]: a susceptible tooth structure (host), a diet rich in fermentable carbohydrates, and cariogenic microbial flora. Additionally, the primary source of *S. mutans* in infants during the first 12-48 months is typically the mother. The risk of transmission increases if the mother has poor oral hygiene or frequent snacking habits, while early exposure to sugar further elevates *S. mutans* levels, increasing the likelihood of dental caries [21]. While *S. mutans* initiates the disease, *Lactobacilli* play a significant role in its progression. Prolonged exposure of dental surfaces to fermentation acids leads to demineralisation, ultimately contributing to cavity formation [22]. ECC is associated with many other risk factors, such as environmental factors, the presence of caries in the mother, lack of fluoride, poor oral habits, feeding practices, frequent consumption of cariogenic food, and night-time bottle feeding [9].

Cariogenic microorganisms

Due to an imbalance in the microbial community present in the oral cavity, particularly on the tooth surface, caries lesions occur as the microorganisms responsible for caries become more active [23]. Various species are responsible for the initiation and progression of the disease, including *S. mutans*, *Streptococcus sobrinus*, *Lactobacilli*, *Actinomyces gerencseriae*, *Bifidobacterium* species, *Candida albicans*, and a few non-mutant species [23]. The most commonly associated species are *S. mutans* and *S. sobrinus*. The organism that initiates the carious process is mainly *S. mutans* [24]. *S. mutans* is involved in metabolising sugars to produce acids on the tooth surface, which results in demineralisation. The increased number of caries corresponds to high levels of *S. mutans* [9]. *A. gerencseriae* is also associated with the initiation of carious lesions, similar to *S. mutans*. In deep caries lesions, *Bifidobacterium* is often identified. In the pathogenesis of carious lesions, *C. albicans* also plays a vital role. In addition, other bacteria present in dental plaque may be linked to the advancement of dental caries [25]. 

Diet

Diet is one of the most important causes of dental caries, among various other factors. Sugar remains one of the major dietary contributors [26]. Modern dietary patterns often include a wide range of fermentable carbohydrates, such as foods containing novel synthetic carbohydrates, refined sugar, and highly processed starch [27]. A child is at higher risk of dental caries if the diet contains high levels of fermentable carbohydrates [9]. Additionally, if the duration of exposure to these fermentable carbohydrates is prolonged, it may aggravate the risk of ECC [9]. Despite being the strongest bony structure, the formation of teeth is significantly impacted by the diet and metabolism of the foetus immediately after birth [28]. Body weight is also a factor, as several studies suggest that children with low body weight are more prone to caries than children with average body weight, due to abnormal calcification of teeth during formation [28]. Certain feeding habits are also involved in the onset of ECC, including bottle feeding at bedtime or while sleeping (which may trigger the disease), recurrent exposure to sugar, consumption of sweetened drinks, and sticky, sugary foods. Maternal nutritional status and caries status, along with oral hygiene habits, have a significant impact on ECC [9]. 

Sugar

One of the major key factors responsible for causing ECC is sugar [29]. Eating patterns that include high amounts of sugar often lead to the early prevalence of dental caries [30]. The demineralisation of the tooth structure is caused by the metabolisation of sugars by the bacteria involved in biofilm formation, which in turn results in the generation of acidic by-products. Sucrose is more cariogenic than any other sugar and, for this reason, is known as the “arch-criminal.” Sucrose has a unique ability to enhance the accumulation and synthesis of extracellular glucans by *S. mutans* in dental plaque [31]. According to a study, children who were exposed to sugar before 12 months had 38% more cavitated lesions compared to those who received sugar after 24 months of age. Additionally, children introduced to early sugar intake had a 29% higher prevalence of caries than those introduced to sugar after 24 months [21]. The World Health Organization advocates exclusive breastfeeding for the first six months of life. Following this period, non-exclusive breastfeeding is recommended for children from six months up to two years of age. Healthy foods should be introduced into a child’s diet after the age of six months [32]. According to the American Heart Association and the IAPD, offering free sugars to children under two years of age should be restricted to reduce sugar consumption during the formative years [33]. Moreover, the WHO advises that free sugar consumption should be restricted to under 10% of total energy intake for both children and adults [34]. This recommendation aims to reduce the likelihood of non-communicable diseases and enhance overall health. Despite the introduction of these guidelines, the prevalence rate of caries remains high among children worldwide, ranging from 44% to 98% in the first year of life [1]. 

Feeding practices

Feeding practices have a significant role in the development of ECC. Feeding practices like breastfeeding and exposing the child to free sugars, including those found naturally in fruit juices or honey in infancy, along with how often they are consumed, significantly influence the onset of ECC [35]. Mothers have a huge impact on children, as they are the first promoters of oral health [36]. Also, they are primarily involved in the dietary habits and food choices of the child. The occurrence of caries was notably high among individuals who were breastfed for extended periods, particularly during the night; those who fell asleep with a bottle; and those whose milk was sweetened with extra sugar [37]. Feeding practices include frequent bottle feeding or breastfeeding more than three times a day, nocturnal bottle feeding or breastfeeding, frequent snacking, feeding a child for too long, giving a child food or beverages that are rich in sugar, low fruit and vegetable intake, and the use of sweetened pacifiers [38]. The onset of ECC is also influenced by when weaning foods are introduced to children. Infants who were introduced to weaning food in a timely manner (between four and six months old) demonstrated a reduced risk of ECC compared to those whose introduction was delayed [39].

Socio-economic status

The high socio-economic status of dental caries is substantially associated with a lower prevalence rate of dental caries [40]. Socio-economic status, which encompasses key elements such as income levels, education, and employment stability, plays a critical role in shaping an individual's access to healthcare services. It also influences diet choices and the extent of health-related knowledge one possesses. For instance, higher income often correlates with better access to nutritious foods and quality dental care, and individuals with higher education levels may be more informed about the importance of oral hygiene practices. Consequently, these factors are vital for maintaining good oral health and preventing dental issues, highlighting the need to address socio-economic disparities in health resources and education [41]. According to a recent study, children in private schools - often coming from more privileged backgrounds - tend to experience lower rates of dental caries compared to their peers in public schools, who generally come from less advantaged socio-economic situations [42]. Children from food-insecure backgrounds frequently exhibit a higher prevalence rate of caries; this can further complicate the relationship between the high occurrence rate of caries and underprivileged populations [43]. Parental traits, such as education and habits, significantly affect children's oral health. Studies show that a mother's education and a family's income level play important roles in shaping how children care for their teeth and overall oral health [44]. Children learn consistent behavioural habits at home, with parents - especially mothers - playing a crucial role in their oral health. For younger children, the involvement of parents and caregivers is vital in preventing dental caries. Key factors such as parental education, attitudes, and beliefs significantly influence how parents manage their children's oral health. Studies show that higher parental education leads to better oral health outcomes for children. Families with lower education levels often neglect dental care and routine visits to dental professionals, increasing the risk of dental caries [45].

Environmental factors

ECC is a combination of various factors that predominantly include lack of awareness, lack of parental education, improper feeding practices, low socio-economic background, deficiency of access to proper dental treatment, and poor oral hygiene habits. For the development of speech, eating, self-esteem, and a positive image in children, good oral health plays a significant role [9]. Contaminated water also plays a role in caries. There is limited understanding of how unsafe water and poor sanitation are related to ECC, even though caries is classified as a hygiene-related illness. Research has shown that perfluorodecanoic acid, an industrial surfactant present in drinking water, might interfere with enamel development and raise the risk of developing ECC [46]. Air pollution consists of deadly matter and ambient particulates that are associated with various systemic diseases [47]. One study suggests that, based on the severity of exposure to carbon dioxide-contaminated air, changes in the composition of saliva and teeth are noticed [48]. Saliva serves as the main defence system, as it plays a protective role against the growth of dental caries. Various factors in saliva help to reduce the formation of dental caries, including flow rate, buffering capacity, saliva pH, antimicrobial properties, and the clearance of food from the oral cavity. Low saliva flow at night increases the risk of tooth decay from high-sugar foods in children and infants [9]. Certain prenatal conditions, such as low birth weight, premature birth, malnutrition, and illness, are related to the presence of enamel hypoplasia [49]. This results in hypoplasia-associated S-ECC. This type of caries is usually seen in young children living in or below poverty. It is indicated by primary teeth that are structurally damaged and vulnerable to caries [50].

Clinical presentation

ECC is an aggressive form of tooth decay that can be distinguished into early, moderate, and late decay [9]. It develops over time and is difficult to diagnose in the early stage. Tooth decay can be observed as follows: initially, a dull white band or spot on the tooth surface near the gum line, which is often undetected by parents; progression of decay is indicated by a yellow, brown, or black band on the tooth surface; and brownish-black stumps indicate advanced decay [4].

Due to ECC, children suffer from pain and difficulty in eating and talking. In advanced cases, extraction of teeth is often essential. This can result in developmental delays involving speech articulation and patterns [9]. Various efforts have been made to create a classification system for ECC (Tables 1-3) [9,51,52].

**Table 1:** Classification based on the severity of ECC and etiology Table credit: [9] ECC, Early Childhood Caries

Type	Classification
Type 1	The existence of isolated carious lesion(s) involving incisors and/or molars. The most common causes are usually a combination of semisolid or solid food and lack of oral hygiene.
Type 2	Early childhood caries (ECC) was described as "labiolingual lesions" affecting maxillary incisors, with or without molar caries, depending on the age of the child and the stage of the disease. Typically, the mandibular incisors are unaffected. The cause is usually the inappropriate use of a feeding bottle, at-will breastfeeding, or a combination of both, with or without poor oral hygiene.
Type 3	ECC was described as carious lesions affecting almost all teeth, including the mandibular incisors. A combination of cariogenic food substances and poor oral hygiene is the cause of this type of ECC.

**Table 2:** Classification based on patterns Table credit: [51]

Type	Classification
Type 1	Lesions associated with developmental defects (pit and fissure defects and hypoplasia)
Type 2	Smooth surface lesions (labial-lingual lesions, approximal molar lesions)
Type 3	Rampant caries - having caries in 14 out of 20 primary teeth, including at least one mandibular incisor

**Table 3:** Classification based on ECC and S-ECC by the participants at the workshop in Bethesda, 1999 DMFS, Decayed Missing Filled Surfaces; DMF, Decayed Missing Filled Teeth; ECC, Early Childhood Caries; S-ECC, Severe Early Childhood Caries Table Credit: [52]

Age	Early childhood caries	Severe early childhood caries
<12	1 or more DMFS surface	1 or more smooth DMF surface
12-23	1 or more DMFS surfaces	1 or more smooth DMF surface
24-35	1 or more DMFS surfaces	1 or more smooth DMF surface
36-47	1 or more DMFS surfaces	1 or more cavitated, filled, or missing (due to caries) smooth surfaces in primary maxillary anterior teeth or DMFS score >4
48-59	1 or more DMFS surfaces	1 or more cavitated, filled, or missing (due to caries) smooth surfaces in primary maxillary anterior teeth or DMFS score >5

Diagnosis

ECC is a serious condition that affects preschool children; it should be diagnosed quickly to optimise its management. Key signs include cavitated lesions, discoloured enamel, and breaks in plaque [29]. Important risk factors include a poor diet, improper oral hygiene, and social influences [53]. Caries-risk assessments are crucial to accurately determine the risk of ECC. The subsequent improvements in diagnostic technology allow for the early detection of ECC. Such timely interventions may avoid more invasive treatments, thus relieving the need for more invasive and conservative techniques in the management of dental caries [54].

ECC is initially identified as a dull, white appearance of de-mineralised enamel that rapidly progresses to noticeable decay along the gum line [55]. The decay is typically first observed on the primary maxillary incisors, and it is common for all four of the maxillary anterior teeth to be affected simultaneously [6]. Carious lesions can occur on either the labial or lingual surfaces of the teeth and, in certain instances, present on both. A yellow or brown cavitated spot visibly marks the decayed hard tissue [56]. In older children with fully erupted primary teeth, it is not uncommon to observe significant deterioration of dental health [57].

The term S-ECC is used instead of rampant caries [6] when specific criteria are met, including any indication of caries on a smooth surface in children under three years old, decay, missing (due to caries), or filled smooth surfaces of an anteroposterior primary tooth in children aged 3-5 years, or a DMFT index equal to or exceeding 4 at age 3, 5 at age 4, and 6 at age 5. Diagnostic aids commonly used for detecting ECC include laser fluorescence and electric caries meters. Additionally, recent non-invasive techniques, such as Quantitative Light-induced Fluorescence, DIAGNOdent, Fibre-optic Transillumination, and Electrical Conductance, have been introduced. The use of bitewing radiographs further enhances the detection of interproximal surface caries, revealing a significant number of carious lesions and inadequate restorations [58].

Management

The primary dentition of a child should be well-maintained for the healthy condition of child. Primary dentition is important for sustaining esthetics, phonetics, mastication, space maintenance, and preventing habits [59]. Prevention of ECC can be accomplished by various means, such as reducing a sugar-rich diet, maintaining good oral hygiene, educating parents, and utilising preventive agents like fluorides [9]. Fluoride varnishes applied by professionals and the use of mouth rinses containing fluoride have also illustrated a decrease in childhood dental cavities. Given that treatment for ECC is both costly and associated with health risks, along with the high recurrence rate following such treatments, the focus has shifted towards primary prevention [60]. The Centers for Disease Control and Prevention defines the three levels of prevention as follows [61]: (I) primary prevention: prevention that occurs before the presence of disease; (II) secondary prevention: identifying the disease before symptoms begin to be noticed; (III) tertiary prevention: steps taken to hinder the disease progression.

Preventing ECC should begin with educating parents during pregnancy and continue throughout the perinatal period, including after birth, for both the mother and child. Management of ECC can occur at home, professionally, and within the community [9].

The home-based treatment of ECC includes prevention and early intervention [9]. Developing good oral hygiene by brushing your child's teeth twice a day with fluoride toothpaste, beginning as soon as the first tooth comes in, is most important [62,63]. Restricting sugary foods and beverages, particularly at bedtime, to avoid decay; use water instead of sugary liquids in bottles [64]. A diet that is high in vitamin D and calcium supports healthy teeth, and the use of fluoride toothpaste assists in the hardening of enamel [65]. Frequent dental visits from age 1 are essential for early detection and professional cleaning [66]. Home care may control early decay, but visiting a dentist is necessary if the situation becomes severe [67]. Early habits, such as brushing and dietary selection, prevent and control ECC [68].

Professional ECC management entails early detection, diagnosis, and treatment by a pediatric dentist [69]. Treatment can consist of restorations, placing crowns over carious teeth to seal caries and prevent progression, or even extractions in advanced cases [70]. The most common preventive measures include sealant application to pits/fissures and fluoride application on smooth tooth surfaces [71]. Parental counselling is essential for the prevention of ECC; it should be done at a suitable age. Routine dental examinations, usually every 6-12 months, provide continuous surveillance and prevention of future dental complications [72]. Dentists suggest effective caries prevention strategies, including xylitol, amorphous calcium phosphate, silver diamine fluoride (SDF), and systemic fluoride [73]. Behavioural approaches like oral health assessments and motivational interviewing also help improve oral hygiene practices [74].

Community-based Early Childhood Care and Education (ECCE) guarantees quality education and care for young children in the community [75]. Community support is mobilised through campaigns and workshops, parental involvement is encouraged, and accessible ECCE centres with trained caretakers are put in place [76]. Health and nutrition support, safety standards, and child protection policies are critical. Partnerships with NGOs and the local government are vital for resources and finances. Involving the community in ECCE care is a foundation for long-term success for children [77].

Role of fluoride

**Figure 1:**
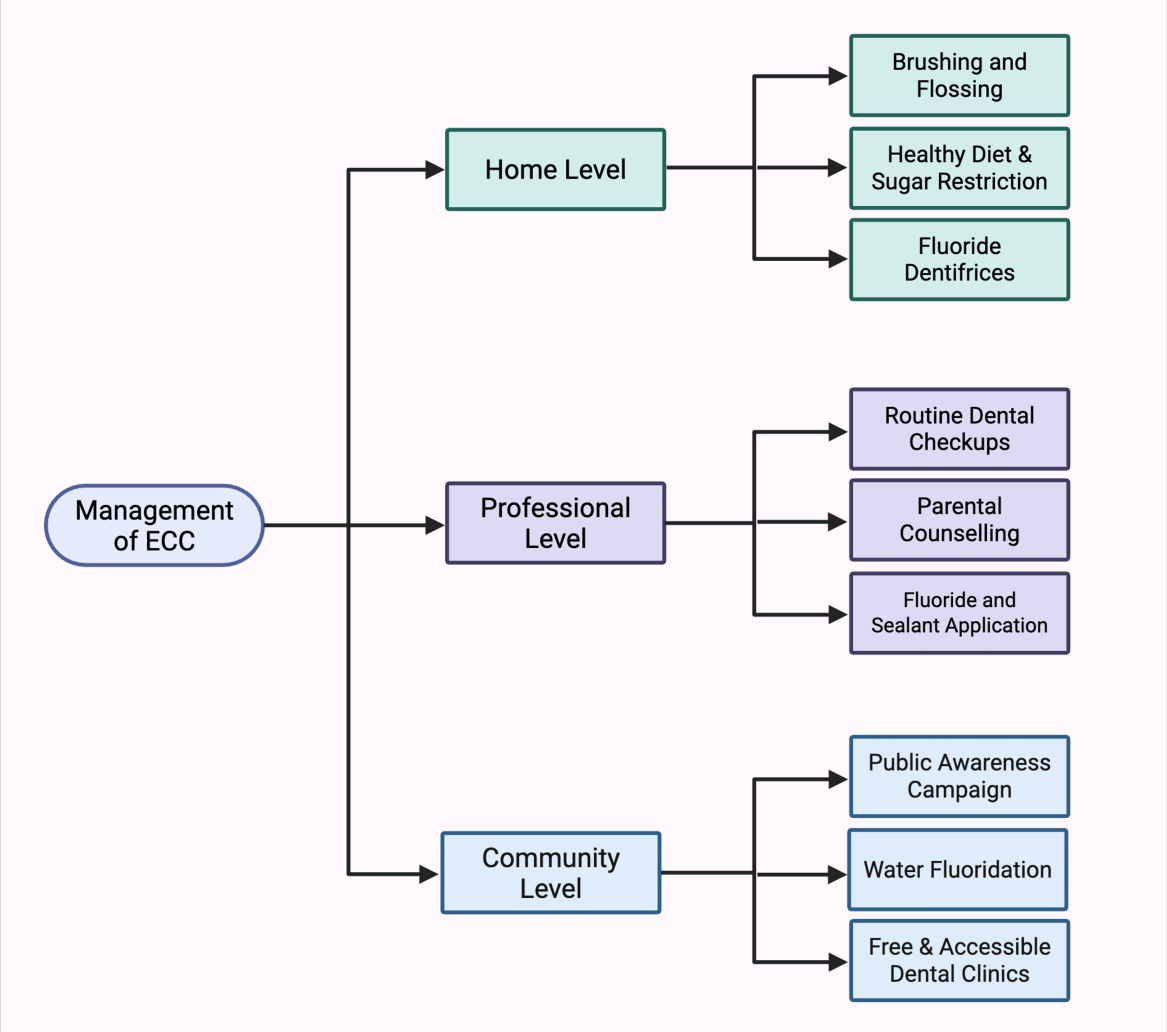
Management strategy of ECC This figure was drawn using the premium version of BioRender [86] (https://BioRender.com/2isqgzi), accessed on April 30th, 2025, with license number YL287LTLH2. Image Credit: Vishnu Desai ECC, Early Childhood Caries

Fluoride is essential for the prevention and control of ECC, a common dental problem in young children. Fluoride fortifies tooth enamel by remineralising decayed spots caused by acids formed by pathogenic bacteria, thereby making enamel more resilient to subsequent acid attacks from sugary foods and beverages [78]. Recent advancements in fluoride treatments have enormously improved caries prevention, particularly for children [79]. One of the most significant advances is SDF, a liquid that is applied directly to affected areas to stop the advancement of early caries without resorting to invasive treatments [80]. SDF remineralises enamel and forms a protective coating, and it is particularly beneficial for children who might have difficulty with conventional dental treatments [81]. Another important development is the application of fluoride varnishes, which are stronger than standard fluoride treatments. These varnishes are enamel-bonding, slowly releasing fluoride, and provide protection for a long time [82]. In addition, fluoride-releasing restorative materials like sealants and fillings continue to provide fluoride protection as they protect teeth from additional harm [83]. Newer fluoridated toothpaste compositions now have better bioavailability and, thus, better enamel absorption [84]. All these innovations in fluoride treatments, in addition to oral hygiene and eating habits, present good ways of preventing and managing ECC, leading to long-term oral health in children (Figure 1) [85,86].

Policy recommendations

The AAPD recognises ECC as a chronic infectious disease influenced by multiple factors over time and emphasises the importance of evidence-based preventive strategies. To mitigate the risk of ECC, the AAPD recommends avoiding frequent consumption of sugary liquids and foods - particularly sugar-sweetened beverages such as juices, soft drinks, sports drinks, and sweetened teas - as well as limiting prolonged breastfeeding after tooth eruption and nursing bottle use beyond 12-18 months. Oral hygiene practices should begin with the eruption of the first tooth, with parents brushing their child’s teeth twice daily using an age-appropriate soft-bristled toothbrush and fluoridated toothpaste - using a smear or rice-sized amount for children under three, and a pea-sized amount for those aged 3-6. The AAPD also advocates for professionally applied fluoride varnish treatments for children at risk of ECC, the establishment of a dental home by age one for early risk assessment and parental education, and collaboration with medical providers to ensure access to dental screenings and preventive care for infants and toddlers. Additionally, the AAPD highlights the importance of educating legislators, policymakers, and insurers on the impact of ECC and the value of prevention [87].

Limitations of this study

This review article has several limitations that should be acknowledged. Firstly, there is a geographic and socioeconomic bias, as most of the research included is localised to specific regions, limiting the ability to generalise findings on a broader scale. Additionally, a lack of long-term data is evident, as many studies focus on short-term effects while overlooking the long-term impact. The study also faces limitations in data availability, as the database used may not encompass all relevant literature on ECC. Lastly, there is a publication bias, as the review only includes literature published in English, thereby excluding valuable research conducted in other languages.

## Conclusions

Despite being a preventable disease, ECC is one of the major oral health problems in the world, even in developed countries. Many risk factors are involved in its initiation and progression, especially for children from deprived populations. ECC can begin as soon as the first tooth erupts in the oral cavity. If not treated completely, it can affect both primary and permanent dentitions. It can affect mental health, learning ability, self-esteem, and normal activities. ECC can cause pain, discomfort, infections, and distress to children. The condition can lead to social and psychological challenges, impacting a child's overall well-being. The presence of visible tooth decay can diminish a child's self-esteem, as they may feel self-conscious or embarrassed about their appearance. These feelings can hinder social interactions and undermine their confidence.

Prevention and management of caries should be done to improve the quality of life of children. Enhancing oral health literacy among the public is essential, and cooperation among other healthcare professionals should be established for the early detection of caries. To minimise the risk factors, it is essential to follow the policies set forth by the AAPD. Hence, oral health promotion and prevention of ECC should be prioritised while promoting general health.
